# The developmental remodeling front: resorptive cell–vascular coupling at the chondro-osseous junction

**DOI:** 10.3389/fcell.2026.1942780

**Published:** 2026-09-16

**Authors:** Haocheng Zhang, Huiying Li, Zhe Li, Peng Yu, Yuanbo Ding, Dongfang Meng, Xiangli Zhang, Jiabao Song

**Affiliations:** 1 Department of Orthopedics I, The First Affiliated Hospital of Henan University of Chinese Medicine, Zhengzhou, China

**Keywords:** angiogenesis, cartilage remodeling, chondro-osseous junction, endochondral ossification, growth plate, osteoclast, primary spongiosa, septoclast

## Abstract

The chondro-osseous junction is commonly described as a site where cartilage is removed and bone is deposited, but this sequence does not explain how matrix topology is relayed into primary trabecular architecture. This review frames the junction as a developmental remodeling front in which cartilage-derived signals, endothelial advance, matrix-processing cell states, temporary scaffold retention, and osteoid deposition overlap in space and time. Mouse perturbation studies support vascular endothelial growth factor A (VEGF-A)-dependent entry, endothelial matrix metalloproteinase 9 (MMP-9)-mediated cartilage processing, endothelial Delta-like ligand 4 (Dll4)–Notch support of mesenchymal septoclasts, and state-specific interactions between endothelium and osteoclast-lineage cells. Recent evidence further shows that osteoclast-supporting receptor activator of nuclear factor-κB ligand (RANKL) sources change during development and that hypertrophic-chondrocyte Piezo1 modulates the local RANKL/osteoprotegerin (OPG) balance, arguing against a single, stage-invariant source model. The evidence nevertheless does not yet quantify the relative proteolytic contributions of endothelium and septoclasts or demonstrate that their timing directly specifies three-dimensional trabecular topology. Osteopetrosis, hypophosphatemic rickets, and collagen-X chondrodysplasia illustrate how similar cartilage-retention phenotypes can arise from distinct initiating compartments. A decisive test will require reciprocal, temporally restricted perturbations, activity-resolved imaging, anatomically matched three-dimensional reconstruction, and parallel adjacent-layer rescue. The remodeling-front framework therefore functions as a causal localization model and a set of falsifiable predictions rather than a single established pathway.

## 1 Introduction

Skeletal morphogenesis and remodeling are often framed as temporally distinct: developmental programs establish bone shape, whereas remodeling maintains the mature skeleton. Endochondral ossification blurs this boundary. During long-bone development, hypertrophic cartilage is mineralized and selectively removed as vessels invade and bone is deposited at the chondro-osseous junction (Gerber et al., 1999; Mackie et al., 2008). Growth, matrix turnover, and architectural refinement therefore converge at this moving interface.

Mineralized cartilage is not cleared completely before osteogenesis begins. Transverse barriers are opened, whereas longitudinal cartilage septa persist as temporary scaffolds for bone deposition (Lee et al., 1995; Mackie et al., 2008; Amizuka et al., 2012). Human growth-plate studies document vascularization of primary and secondary ossification centers (Walzer et al., 2014). In mouse bone, septoclasts localize beside endothelial buds (Bando et al., 2018), endothelial proteolysis and vessel-associated osteoclast states operate at the advancing front (Romeo et al., 2019), and mesenchymal septoclasts participate in developmental cartilage resorption (Sivaraj et al., 2022). Primary trabeculae thus emerge through a selective matrix handoff rather than the sequential replacement of one tissue by another.

Perturbation studies reveal that this handoff is coupled across cell compartments. Blocking vascular endothelial growth factor A (VEGF-A) suppresses capillary invasion, chondroclast recruitment, cartilage removal, and trabecular formation, whereas *Mmp9* loss delays vascularization and ossification (Vu et al., 1998; Gerber et al., 1999). More resolved mouse studies show that endothelial proteolysis contributes to cartilage removal and directional elongation, endothelial Delta-like 4 (Dll4) supports mesenchymal septoclast specification, and non-bone-resorbing vessel-associated osteoclasts regulate type H vessel anastomosis (Romeo et al., 2019; Sivaraj et al., 2022). These findings establish reciprocal dependencies but assign matrix degradation and vascular patterning to different cell states. The unresolved problem is therefore not whether resorption and angiogenesis co-occur, but how endothelial, septoclast, and osteoclast-lineage activities are partitioned and synchronized.

We define the chondro-osseous junction as a developmental remodeling front whose output depends on the relative timing of matrix opening, vascular advance, scaffold retention, and osteoid deposition. The principal scope of this review is primary endochondral ossification in mouse long bones, extending from embryonic primary-ossification-center formation through postnatal remodeling at the growth plate and primary spongiosa. Human histological and ultrastructural observations are used to establish anatomical and disease relevance, whereas secondary ossification centers are considered only as explicitly identified comparative contexts. Evidence from fracture repair, adult bone modeling or remodeling, ovariectomy-related models, and other nondevelopmental settings is used only to nominate mechanistic analogues and is not treated as direct evidence for the developing remodeling front. Within these boundaries, this review maps the evidence for the framework, distinguishes direct developmental perturbation from spatial inference and cross-context extrapolation, and asks when mistimed coordination produces a transient delay versus a persistent architectural defect.

## 2 The developmental remodeling front is a matrix-defined multicellular interface

The chondro-osseous junction is often described by its resident cell types, but its developmental function is better understood by first defining the matrix problem those cells must solve. The front must open a path through terminal hypertrophic cartilage without erasing the longitudinal scaffold on which primary bone is deposited. Matrix removal is therefore selective rather than uniform, and the relevant cellular categories are partly determined by substrate, position, and timing. This section accordingly defines the matrix topology and participating cellular states—who is present, where each state is located, and which substrate it is positioned to process—while Section 2 addresses the directional signals that coordinate them.

### 2.1 Matrix topology sets the architectural problem

Hypertrophic cartilage is spatially heterogeneous before vascular entry. Mineralization is concentrated in the longitudinal intercolumnar septa, whereas transverse partitions are less extensively mineralized. At the advancing front, transverse septa are breached as capillaries enter, while longitudinal calcified cartilage cores persist and become coated with osteoid (Lee et al., 1995; Amizuka et al., 2012). The earliest trabeculae are therefore composite structures: a cartilage core records the geometry of the growth plate, and newly deposited bone stabilizes that temporary scaffold (Mackie et al., 2008; Amizuka et al., 2012). The relevant developmental output is not complete cartilage removal, but conversion of an anisotropic cartilage template into an ordered cartilage–bone network.

This distinction separates physiological retention from pathological persistence. Cartilage within primary trabeculae is expected during normal ossification; the informative variables are how much remains, whether it is longitudinally oriented and connected, and how long it persists before secondary remodeling. Osteopetrotic models illustrate the consequence of disturbing this balance, with broad cartilage plates and disorganized trabeculae replacing the narrow remnants found during normal ossification (Josephsen et al., 2009).

This topology also argues against treating resorption as a single reaction. Opening transverse barriers, removing mineralized cartilage, clearing cellular and matrix debris, and preserving longitudinal cores impose different biochemical and mechanical demands. Histology establishes their spatial sequence, but it cannot by itself assign each task to a unique cell population. Functional attribution therefore requires combining location with lineage dependence and perturbation evidence.

### 2.2 Multinucleated resorptive cells occupy a mineralized-substrate state within the osteoclast lineage

Multinucleated, tartrate-resistant acid phosphatase (TRAP)-positive cells at the terminal growth plate share the major morphological and molecular machinery of bone-resorbing osteoclasts. When apposed to calcified cartilage, they are commonly termed chondroclasts. This term is anatomically useful but should not be treated as evidence for an independent lineage. In this review, *chondroclast* refers to a multinucleated osteoclast-lineage cell actively apposed to mineralized cartilage, whereas *osteoclast* is retained as the broader lineage and functional term.

This substrate-based definition accommodates specialization without assuming developmental separation. Transcriptomic comparison of laser-captured chondroclasts and osteoclasts from mouse fracture callus identified distinct molecular signatures and regulatory networks (Khan et al., 2020). The result supports context-dependent specialization, but the repair setting, sampling strategy, and absence of lineage tracing prevent extrapolation to two fixed clast lineages in the developing growth plate. The more defensible interpretation is that mineralized cartilage can elicit a specialized osteoclast-lineage state whose stability and reversibility remain unresolved.

Classical osteoclast-lineage cells facilitate, but do not uniquely execute, opening of the developing marrow cavity. In embryonic *Rank*-deficient mice, cavity formation persisted despite the absence of osteoclasts, although it was delayed and structurally abnormal (Tosun et al., 2022). Thus, multinucleated clasts are well positioned to remodel mineralized cartilage and later bone, but their presence should not be used to assign all cartilage removal, debris clearance, or vascular advance to a single cell class.

### 2.3 Septoclasts define a mesenchymal, vessel-associated cartilage-processing state

Septoclasts are distinguishable from multinucleated clasts by morphology, lineage, and substrate position. Initially identified in young rat growth plates, these mononuclear cells contain cathepsin B-rich vesicles and extend processes between invading capillaries and terminal transverse septa (Lee et al., 1995). Mouse studies subsequently localized fatty acid-binding protein 5 (FABP5)-positive septoclasts beside endothelial buds and showed expression of perivascular markers (Bando et al., 2018). Their anatomy is therefore consistent with processing non- or poorly mineralized cartilage immediately ahead of, or adjacent to, the advancing vessel rather than acid-dependent resorption of mineralized longitudinal cores.

Lineage studies now provide a stronger identity framework than marker localization alone. Inducible *Pdgfrb*-lineage tracing, together with the absence of *Vav1*-lineage labeling, identified FABP5-positive septoclasts as mesenchymal stromal derivatives rather than hematopoietic osteoclasts; these cells contained chondrocyte-derived material and expressed a cartilage-processing program (Sivaraj et al., 2022). Fetal mapping further identified CD55^+^CD90^+^ interface mesenchyme as a transient source of septoclasts during perinatal development (Sumiya et al., 2026).

Septoclast identity should nevertheless remain operational rather than marker-exclusive. FABP5, perivascular location, and a protease-rich phenotype can enrich for the state, but none alone proves that a cell performs the local matrix-opening event. The strongest current definition therefore combines mesenchymal ancestry, adjacency to endothelial buds, uptake of cartilage-derived material, and expression of a cartilage-processing program. How this state is maintained and how much matrix removal it contributes are causal questions reserved for Section 2.

### 2.4 Hypertrophic cartilage is an active cellular compartment and a source of osteogenic descendants

The front cannot be divided into degradative cells on one side and imported bone-forming cells on the other. Hypertrophic chondrocytes construct and mineralize the substrate, supply local regulatory cues, and in mouse lineage-tracing studies generate descendants that cross the cartilage-to-bone boundary and enter osteogenic lineages in the primary spongiosa (Yang et al., 2014; Zhou et al., 2014). These experiments establish ancestry and osteogenic contribution in the tested models, but they do not determine whether the transition is direct, passes through a progenitor-like state, or contributes equally across ages and skeletal sites. “Chondrocyte-derived osteogenic cells” is therefore more precise than assuming universal direct transdifferentiation.

Osteoid deposition at the front may consequently involve both perivascular osteogenic progenitors and chondrocyte-derived descendants. Their coexistence matters because successful matrix opening and vessel occupancy do not themselves generate a trabecula: an osteogenic cell must reach and coat the retained longitudinal core. Whether either osteogenic source is preferentially positioned by cartilage geometry or vascular trajectory remains unresolved, but both belong to the cellular definition of the remodeling front rather than to a later, separate phase.

### 2.5 Non-osteoclast macrophages broaden the cellular boundary of the front

The immune compartment is broader than the osteoclast lineage. Bone-resident macrophages, often termed osteomacs, are mononuclear phagocytes distinct from multinucleated TRAP-positive osteoclasts and from mesenchymal septoclasts. Their potential clearance, osteogenic support, and vascular functions are considered in Section 2; at the level of identity, the essential point is that broad myeloid markers or depletion strategies cannot distinguish osteomacs from osteoclast precursors and specialized vessel-associated osteoclast states.

Together, these observations define a matrix-organized interface containing at least five functionally distinct components: mineralized-substrate osteoclast-lineage cells, vessel-associated mesenchymal septoclasts, hypertrophic chondrocytes and their descendants, endothelial and perivascular cells, and non-osteoclast macrophages. This cellular map answers who is present and which local task each state is positioned to perform, but it does not establish who instructs whom. Section 2 therefore shifts from identity and localization to perturbation-defined dependencies, asking how angiogenic entry, proteolysis, osteoclast support, vascular organization, and biomechanical context are coordinated as a partitioned circuit.

## 3 Directional perturbations reveal a partitioned resorptive–vascular circuit

Colocalization at the chondro-osseous junction does not establish coupling. A vessel and a matrix-processing cell may respond independently to hypertrophic cartilage, one may deliver or instruct the other, or both may participate in rapid feedback. The available perturbations support several directional links, but they do not resolve into one linear pathway. Instead, cartilage-derived signals, matrix presentation, endothelial activity, septoclast state, osteoclast-supporting niches, and local mechanical conditions appear to divide control across developmental stages and substrates (Table 1).

**TABLE 1:** Evidence map for relationships at the developmental remodeling front.

Claim	Species/model context	Evidence design	Direct observation or perturbation result	Supported inference	Boundary or falsifier
VEGF-dependent vascular entry	Postnatal mouse; systemic VEGF blockade	Reversible pharmacological loss-of-function (Gerber et al., 1999)	Capillary invasion, clast recruitment, cartilage removal, and trabecular formation were reversibly suppressed together	VEGF signaling is required for coordinated entry and ossification in this model	Systemic blockade does not order the downstream events or identify a responding resorptive cell
Matrix presentation of VEGF	Mouse; postnatal Hspg2-deficient endochondral bone	Genetic loss-of-function with cartilage-targeted perlecan rescue (Ishijima et al., 2012)	Perlecan restoration rescued growth-plate vascularization and supported endothelial VEGF-receptor activation	Ligand presentation and matrix context condition VEGF effectiveness	Perlecan also changes matrix properties independently of VEGF
Endothelial proteolysis	Mouse; postnatal long-bone endothelium	Endothelial-specific Mmp9 loss-of-function (Romeo et al., 2019)	Cartilage processing and growth-plate remodeling were altered	Endothelial MMP-9 contributes directly at this interface	MMP-14 was expressed but not equivalently tested; its quantitative contribution relative to MMP-9 and septoclast-derived proteolysis remains unknown
Septoclast identity and endothelial dependence	Mouse; developmental ossification and fracture-healing models	Pdgfrb/Vav1 lineage tracing plus endothelial Dll4 perturbation (Sivaraj et al., 2022)	FABP5-positive mesenchymal cells contained chondrocyte debris; Dll4 loss reduced their abundance, MMP-9 expression, fragment removal, and long-bone growth	Endothelial Notch signaling supports a cartilage-processing septoclast state	Endothelial deletion is not selective septoclast ablation; unchanged matrix opening after selective septoclast ablation would falsify necessity
Septoclast protease program	Juvenile mouse; primary septoclast culture	Transcriptomics plus Ets1 knockdown in primary septoclasts (Bando et al., 2025)	Ets1 knockdown reduced Ctsb and Mmp13 expression in culture	ETS1 regulates part of the septoclast protease program	Its *in vivo* contribution to cartilage removal and vessel advance remains untested
Developmental RANKL-source relay	Mouse; fetal/perinatal medullary-cavity development	Reporter analysis, single-cell profiling, and fate mapping (Sumiya et al., 2026)	CD55^+^CD90^+^ mesenchyme transiently generated septoclasts and supported early osteoclastogenesis; LepR-positive stroma assumed this support role later	Osteoclast-supporting niches change with developmental stage	The relative RANKL contributions of these sources versus chondrocytes and their direct effects on trabecular topology remain unresolved
Hypertrophic-chondrocyte control of osteoclastogenesis	Mouse; postnatal growth-plate and fracture-repair contexts	Conditional RANKL-, Runx2-, and Piezo1-related genetic perturbations (Xiong et al., 2011; Rashid et al., 2021; Rashid et al., 2024; Tschaffon-Müller et al., 2026)	Perturbations changed the RANKL/OPG balance, osteoclast abundance, cartilage retention, or bone mass	Hypertrophic chondrocytes can tune local osteoclastogenesis in defined postnatal or repair contexts	Cre domains and developmental ages differ; these studies do not establish an exclusive fetal RANKL supply
Osteoclast contribution to cavity opening	Mouse; embryonic marrow-cavity formation	Rank-deficient and related genetic absence models (Tosun et al., 2022)	Marrow-cavity formation persisted but was delayed and structurally abnormal	Osteoclasts facilitate but do not uniquely execute cavity expansion	Developmental compensation and cell-state-specific functions remain unresolved
Endothelial instruction of skeletal cells	Mouse; postnatal long bone	Inducible endothelial Notch disruption with Noggin rescue (Ramasamy et al., 2014)	Vascular growth, osteoprogenitor abundance, chondrocyte maturation, and trabecular formation changed; several endpoints were rescued	Endothelium provides an angiocrine route that supports osteogenesis	The evidence does not show the positioning of osteogenic cells on individual cartilage cores
Osteoclast-state control of vessels	Mouse; postnatal long bone	Cell-state analysis with RANKL–RANK perturbation (Romeo et al., 2019)	Non-bone-resorbing, vessel-associated osteoclasts regulated type H vessel anastomosis	A specialized osteoclast state can control local vascular topology	The downstream RANK mediator and contribution to three-dimensional trabecular topology remain unresolved
Osteogenic sources at the front	Mouse; developmental ossification, postnatal growth, and fracture repair	Histologic synthesis and lineage tracing (Mackie et al., 2008; Yang et al., 2014; Zhou et al., 2014)	Perivascular osteogenic cells and chondrocyte-derived descendants contribute to primary spongiosa bone	Multiple osteogenic sources can coat retained cartilage	Preferential positioning by vessels or cartilage cores and quantitative stage-specific contributions remain unknown
Timed handoff specifies trabecular topology	Predominantly mouse; native growth plate, fracture repair, systemic disease, and secondary ossification	Integrative hypothesis based on spatial histology and discordant perturbation phenotypes (Lee et al., 1995; Vu et al., 1998; Miao et al., 2004; Josephsen et al., 2009; Ota et al., 2009; Amizuka et al., 2012; Romeo et al., 2019; Heilig et al., 2020; Sivaraj et al., 2022)	No study has registered matrix loss, vessel occupancy, osteoid coverage, and final connectivity at the same interface element	Local lead–lag timing may explain why similar cartilage retention produces different architectures	Independent manipulation of event timing without a change in final topology would falsify the strong form of the model

Abbreviations: C, causal perturbation evidence; CTSB, cathepsin B; Dll4, Delta-like ligand 4; FABP5, fatty acid-binding protein 5; LepR, leptin receptor; MMP, matrix metalloproteinase; OPG, osteoprotegerin; P, proposed relationship; RANKL, receptor activator of nuclear factor-κB ligand; S, supported but incompletely isolated relationship; VEGF, vascular endothelial growth factor.

### 3.1 Hypertrophic cartilage and its matrix establish a VEGF-dependent entry module

Hypertrophic chondrocytes initiate vascular entry before physical contact with metaphyseal vessels by producing vascular endothelial growth factor A (VEGF-A). In postnatal mice, soluble VEGF-receptor treatment nearly abolished capillary invasion together with chondroclast recruitment, terminal cartilage resorption, and trabecular bone formation; withdrawal restored vascularization and growth-plate organization (Gerber et al., 1999). The reversibility places VEGF signaling upstream of coordinated entry and ossification in this model. Systemic blockade, however, cannot distinguish a direct effect on endothelial advance from secondary loss of precursor delivery, matrix access, or resorptive-cell recruitment.

The extracellular matrix determines whether VEGF abundance becomes an effective spatial signal. In *Hspg2*-deficient mice, hypertrophic chondrocytes formed and expressed increased *Vegfa*, yet vascular invasion, removal of calcified cartilage, marrow formation, and trabecular formation were severely impaired. Cartilage-specific restoration of perlecan rescued growth-plate vascularization, and biochemical evidence supported perlecan-dependent activation of endothelial VEGF receptors (Ishijima et al., 2012). The causal unit is therefore not VEGF secretion alone, but a cartilage–matrix–endothelial module in which ligand presentation and receptor engagement delimit where an angiogenic cue can be read.

### 3.2 Osteoclast support is distributed through a developmentally relayed RANKL/OPG system

Hypertrophic cartilage also regulates the abundance of osteoclast-lineage cells, but existing studies no longer support a single, stage-invariant source of receptor activator of nuclear factor-κB ligand (RANKL). Conditional skeletal deletions implicated hypertrophic-chondrocyte RANKL in mineralized-cartilage resorption in postnatal mice (Xiong et al., 2011). Hypertrophic-chondrocyte *Runx2* deletion lowered the *Rankl/Opg* ratio, reduced cartilage-degrading enzymes and TRAP-positive chondroclasts, and retained cartilage into the diaphysis (Rashid et al., 2021; Rashid et al., 2024). These perturbations support a local chondrocyte-to-osteoclast route, while differences among Cre domains and ages limit claims of exclusive ligand supply.

Mechanosensitive regulation adds another layer. *Col10a1*-Cre-mediated *Piezo1* deletion increased osteoclast numbers in the primary spongiosa while increasing RANKL and decreasing osteoprotegerin (OPG) expression in hypertrophic chondrocytes, without detectable changes in their apoptosis or osteogenic transition (Tschaffon-Müller et al., 2026). In the tested postnatal and repair contexts, hypertrophic chondrocytes can therefore tune osteoclastogenesis independently of a gross change in chondrocyte survival. This result identifies a mechanosensitive regulatory route but does not establish which physiological force activates it at a particular interface location.

During fetal primary-ossification-center formation, CD55^+^CD90^+^ interface mesenchymal cells were identified as an early RANKL-producing population that supported osteoclastogenesis and transiently generated FABP5-positive septoclasts; later, leptin receptor (LepR)-positive marrow stromal cells assumed this supportive role (Sumiya et al., 2026). RANKL control is thus distributed both across cell types and over developmental time. The relevant causal question is not which single population “owns” RANKL, but how the relative contributions of hypertrophic chondrocytes, interface mesenchyme, and marrow stroma change with age, site, and matrix state.

### 3.3 Matrix proteolysis is distributed, but its quantitative partition remains unresolved

VEGF-dependent entry does not remove the cartilage barrier by itself. *Mmp9*-null mice develop an expanded hypertrophic zone with delayed apoptosis, vascularization, and ossification, and wild-type bone marrow transplantation rescues the phenotype (Vu et al., 1998). This establishes a requirement for a marrow-replaceable MMP-9 source in the timing of entry, but the experiment predates cell-resolved mapping and cannot identify one executing population.

Later studies separate several proteolytic compartments. At the postnatal bone–cartilage interface, endothelial cells expressed several matrix-remodeling proteases at higher levels than osteoclast-lineage cells, with *Mmp9* showing the strongest relative enrichment. Consistent with the previously reported growth-plate phenotype of global *Mmp9* loss, endothelial *Mmp9* deletion directly impaired cartilage processing and growth-plate remodeling, while redirecting angiogenic vessels also changed bone shape (Romeo et al., 2019). Although *Mmp14* was likewise enriched, it was not functionally perturbed in that study, and the reason for its omission was not reported (Romeo et al., 2019). Its independent or compensatory contribution relative to MMP-9 therefore remains an open cell-specific causal gap. FABP5-positive septoclasts expressed MMP-9, MMP-13, MMP-14, and cathepsin B and contained chondrocyte-derived material (Sivaraj et al., 2022). ETS1-dependent regulation of *Ctsb* and *Mmp13* in cultured septoclasts supports a distinct protease program (Bando et al., 2025), whereas soluble epoxide hydrolase inhibition increased endothelial-to-mesenchymal Notch signaling and FABP5/MMP-9-associated septoclast activity together with long-bone growth and fracture repair (Chen et al., 2026). Because the latter intervention modifies endothelial and mesenchymal programs simultaneously, it strengthens pathway plausibility without establishing septoclast-selective sufficiency.

These findings replace a “clast clears, vessel follows” sequence with a substrate- and cell-state-dependent division of labor. Endothelial MMP-9 has direct loss-of-function support at the postnatal interface (Romeo et al., 2019). Mesenchymal lineage tracing and cartilage-fragment uptake establish septoclast positioning and substrate engagement *in vivo* (Sivaraj et al., 2022). ETS1 knockdown supports transcriptional regulation of Ctsb and Mmp13 in cultured septoclasts (Bando et al., 2025). Soluble epoxide hydrolase inhibition provides additional but nonselective pathway evidence for endothelial-to-mesenchymal Notch regulation of septoclast activity (Chen et al., 2026). Classical osteoclast-lineage cells remain positioned to resorb mineralized cartilage and bone, although cavity opening can proceed abnormally and with delay in their absence (Tosun et al., 2022). The available data do not show that these contributions are additive, exhaustive, or constant across developmental stages.

### 3.4 Endothelium is instructive, and its reciprocal partners are cell-state specific

The invading vasculature is not merely a conduit. Inducible disruption of endothelial Notch signaling in postnatal mouse long bone impaired vascular growth, reduced osteoprogenitors and trabecular formation, and altered chondrocyte maturation; recombinant Noggin rescued several skeletal defects (Ramasamy et al., 2014). Endothelial Dll4–Notch signaling also maintained FABP5-positive septoclast abundance and protease expression, whereas endothelial *Mmp9* contributed directly to cartilage processing (Romeo et al., 2019; Sivaraj et al., 2022). These studies assign angiocrine, juxtacrine, and proteolytic functions to endothelium, although no single experiment isolates all three at the same capillary tip.

Endothelial identity and geometry change with developmental stage. Type E endothelium predominates during embryonic and early postnatal osteogenesis and depends on integrin β1–laminin α5 signaling (Langen et al., 2017), whereas type H vessels were defined mainly by their association with osteoprogenitors in postnatal and adult mouse bone (Kusumbe et al., 2014). Neither label alone identifies the endothelial bud that crosses a particular septum. More directly, altered VEGF expression disrupts vascular organization and bone morphology (Ben Shoham et al., 2016), and experimentally misdirected angiogenic vessels produce contorted bone shape (Romeo et al., 2019). Vascular trajectories can therefore carry morphogenetic information, but the mechanism translating a capillary path into primary-trabecular branch orientation remains unresolved.

Evidence for the reverse, resorptive-cell-to-endothelial direction is model-dependent: the principal PDGF-BB data derive from adult bone modeling and ovariectomy-related remodeling rather than developmental primary ossification. In those settings, preosteoclast-derived platelet-derived growth factor-BB (PDGF-BB) increases platelet endothelial cell adhesion molecule 1-high/endomucin-high vessels and bone formation (Xie et al., 2014). This finding provides a mechanistic analogue for reverse coupling but does not demonstrate PDGF-BB signaling from cartilage-apposed chondroclasts during primary spongiosa formation. At the developmental bone–cartilage interface, reciprocal relationships are instead best supported for selected cell states: endothelial Dll4 maintains the septoclast state (Sivaraj et al., 2022), whereas endothelial RANKL–RANK signaling supports non-bone-resorbing vessel-associated osteoclasts (VAOs) that are dispensable for cartilage removal but regulate type H vessel anastomosis (Romeo et al., 2019). “Osteoclast-to-vessel signaling” should therefore not be generalized across resorptive states or developmental contexts.

Non-osteoclast macrophages may provide an additional auxiliary layer. Bone macrophages can clear apoptotic cells through efferocytosis (Sinder et al., 2017), and tissue macrophages promote endothelial tip-cell fusion during developmental angiogenesis outside bone (Fantin et al., 2010). These observations make osteomacs plausible contributors to debris clearance or vessel anastomosis near the front. However, the macrophage-anastomosis evidence is not specific to type H bone vessels, and embryonic long-bone analysis did not identify a major independent macrophage requirement for marrow-cavity formation (Tosun et al., 2022). Direct support for osteomac control of type H anastomosis at the chondro-osseous junction is therefore lacking. Distinguishing osteomacs from VAOs and other CSF1R- or myeloid-marker-positive populations is a necessary knowledge gap rather than a semantic detail.

### 3.5 Local biomechanics may gate the position and timing of biochemical coupling

The remodeling front experiences compression, tension, interstitial fluid movement, and blood-flow-derived shear, yet these forces are rarely registered to the same matrix element as vascular entry or proteolysis. Local stress may therefore act not as a replacement for VEGF, Dll4, RANKL/OPG, or MMP signaling, but as a gate that changes where and when those pathways cross an activation threshold. Matrix deformation could alter the presentation or accessibility of perlecan-bound VEGF (Ishijima et al., 2012), while strain sensed by hypertrophic chondrocytes could modify osteoclast support through Piezo1-dependent RANKL/OPG regulation (Tschaffon-Müller et al., 2026). Flow and tissue strain might likewise influence endothelial tip-cell behavior and the spatial access of endothelial or septoclast proteases, although these links have not been directly tested at the growth-plate front (Romeo et al., 2019; Sivaraj et al., 2022).

Evidence from the adjacent ossification front shows that such a gate is plausible. During periadolescence, mechanical loading activated osteoblast PIEZO1, increased FAM20C-dependent extracellular dentin matrix protein 1, inhibited VEGFR2 signaling, reduced MMP-9 at the ossification-front–growth-plate border, and promoted conversion of growth-associated type H vessels toward quiescent type L endothelium (Dzamukova et al., 2022). Because this model concerns late postnatal vessel maturation rather than initial vascular entry, it cannot be transferred directly to embryonic remodeling. It nevertheless demonstrates that regional loading can couple matrix state to endothelial phenotype. The unresolved test is whether local strain or flow predicts the lag among matrix opening, protease activity, and capillary advance at the same interface element. Until then, biomechanics should be treated as a spatial and temporal modifier rather than an established master regulator.

The resulting model is a set of separable dependencies rather than one reciprocal loop. Cartilage and its matrix permit endothelial entry; different stromal sources relay osteoclast support; endothelial, septoclast, and osteoclast-lineage states divide matrix and vascular tasks; macrophages may provide clearance or vascular assistance; and local forces may shift the thresholds and timing of these interactions. Figure 1 and Table 1 therefore grade individual links rather than implying a single validated pathway.

**FIGURE 1:**
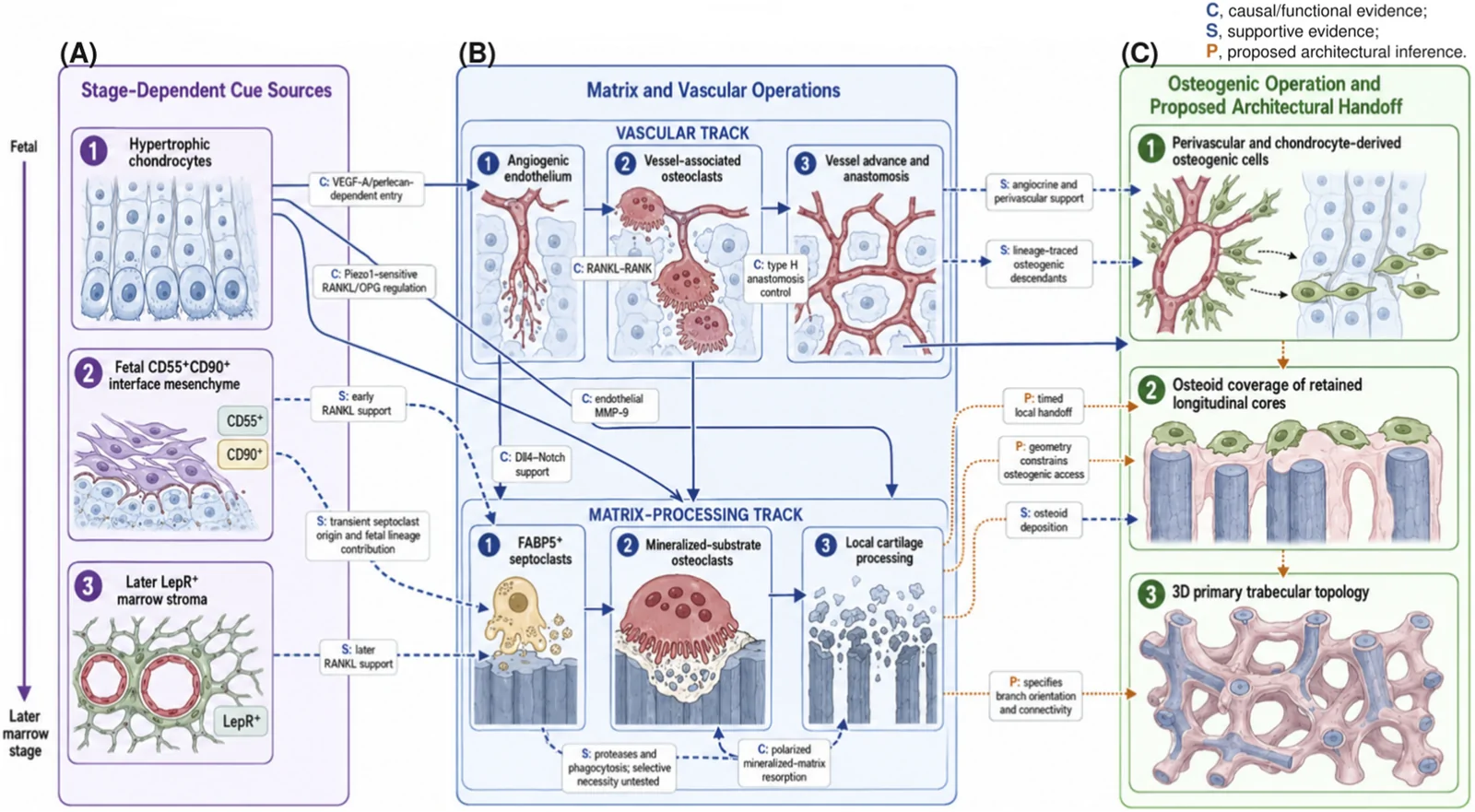
Evidence-graded organization at the developmental remodeling front. **(A)** Stage-dependent cue sources comprise hypertrophic chondrocytes, fetal CD55^+^CD90^+^ interface mesenchyme, and later LepR + marrow stroma. **(B)** Matrix and vascular operations are separated into vascular and matrix-processing tracks involving angiogenic endothelium, vessel-associated osteoclasts, FABP5+ septoclasts, mineralized-substrate osteoclasts, and local cartilage processing. **(C)** The proposed osteogenic handoff links perivascular and chondrocyte-derived osteogenic cells to osteoid coverage of retained longitudinal cores and subsequent three-dimensional primary trabecular topology. Evidence grades apply to each specific directional link in its indicated developmental and model context, rather than to an entire pathway or cell type. C denotes a directional relationship supported by compartment-resolved loss-of-function that isolates necessity for the specific link, or by a mechanism-matched rescue or reversal that isolates the affected causal layer; S denotes evidence that supports the relationship but does not isolate necessity or directionality; and P denotes a framework-derived prediction requiring direct validation. Solid arrows indicate C links, whereas dashed arrows indicate S or P links. Developmental-stage and context qualifiers are shown within the diagram; full details of the corresponding models, results, inferences, and falsifiers are provided in Table 1. Dll4, Delta-like ligand 4; FABP5, fatty acid-binding protein 5; LepR, leptin receptor; MMP, matrix metalloproteinase; OPG, osteoprotegerin; RANKL, receptor activator of nuclear factor-κB ligand; VEGF-A, vascular endothelial growth factor A.

## 4 Coupling kinetics, not resorption alone, shape primary trabecular architecture

Resorptive activity alone does not predict architectural output. Retained cartilage can support abundant cartilage-cored trabeculae when vascular and osteogenic access persists, yet mark failed ossification when that access is lost. Conversely, faster removal need not be harmful if osteoid deposition advances in phase. Architecture therefore depends on the timing among matrix opening, vascular entry, longitudinal-core retention, and bone deposition.

### 4.1 Normal primary trabeculae arise through a timed matrix handoff

Primary trabecular formation begins with asymmetric matrix fate. Endothelial and septoclast proteolytic activity localizes near the advancing vascular front (Romeo et al., 2019; Sivaraj et al., 2022), where transverse partitions are breached, whereas longitudinal calcified septa persist as cores for bone deposition (Lee et al., 1995; Amizuka et al., 2012). These spatial observations are compatible with a local handoff from selective cartilage processing to vascular occupation and osteoid deposition, but no experiment has yet registered all steps at the same matrix element or shown that one population uniquely opens transverse septa. The cartilage core is therefore an observed temporary scaffold; its proposed instructive role in specifying network geometry remains to be tested.

Osteogenic cells entering with vessels contribute to the primary spongiosa (Mackie et al., 2008), while mouse lineage-tracing studies establish an additional contribution from chondrocyte-derived osteogenic cells (Yang et al., 2014; Zhou et al., 2014). Neither body of evidence determines whether these sources are preferentially positioned by cartilage cores or vessel trajectories. Thus, composite early trabeculae and their longitudinal cartilage remnants are well established, whereas the proposed timing rule—that local matrix breach, vessel occupancy, and osteoid coverage must occur within a bounded interval to determine branch length, orientation, and connectivity—remains an integrative hypothesis rather than a demonstrated morphogenetic mechanism.

Local mechanical conditions may help set this interval. In growing mouse long bone, metaphyseal type H capillaries experience higher flow and shear than diaphyseal sinusoids, and acute flow reduction disrupts endothelial budding and anastomosis near the growth plate while lowering endothelial Notch activity (Ramasamy et al., 2016). At the later postnatal ossification front, body-weight-associated mechanical loading activates osteoblast PIEZO1–FAM20C–DMP1 signaling; DMP1 accumulates first in the central front, suppresses VEGFR2 activation, reduces endothelial MMP-9, and promotes the transition from active type H to quiescent type L vessels (Dzamukova et al., 2022). Together with the effect of hypertrophic-chondrocyte Piezo1 on the local RANKL/OPG balance (Tschaffon-Müller et al., 2026), these findings identify plausible force-sensitive inputs into both vascular and resorptive programs. They do not yet show that local stress synchronizes vessel entry with loss of a particular cartilage septum; future pulse studies should therefore register strain or flow, protease activity, and capillary-tip movement at the same three-dimensional matrix element.

### 4.2 Delayed resorption has divergent effects depending on vascular access

Models of impaired turnover show why cartilage retention cannot be interpreted as a uniform increase in scaffold. When VEGF signaling is blocked, or when perlecan deficiency prevents effective endothelial VEGF receptor activation, hypertrophic cartilage persists because vascular entry and associated cell recruitment fail. In the *Hspg2*-deficient growth plate, this arrest produces an almost complete absence of marrow and trabecular bone despite abundant hypertrophic matrix (Gerber et al., 1999; Ishijima et al., 2012). Here, retained cartilage is inaccessible substrate rather than a productive template.

A different outcome occurs when osteogenic deposition continues despite inefficient cartilage removal. Hypertrophic-chondrocyte-specific *Runx2* deletion reduced cartilage-degrading enzymes and osteoclast-supporting signals, decreased osteoclast abundance, and retained cartilage, yet increased adult trabecular mass without increasing osteoblast number, mineral apposition, or bone-formation rate; the bones were mechanically weaker (Rashid et al., 2021; Rashid et al., 2024). The study did not directly quantify how much of the increased mineralized structure was bone deposited on retained cartilage, so that interpretation remains plausible rather than proven. Similarly, defective osteoclast acidification in *Ae2*-null mice produced interwoven trabeculae containing broad cartilage plates and markedly restricted marrow spaces (Josephsen et al., 2009). These models show that more trabecular mineral is not equivalent to more normally organized or mechanically competent bone: bone-volume measurements can conflate altered formation, retained cartilage cores, and failure of subsequent removal.

Disease models reinforce this distinction but introduce additional variables. Hyp mice exhibit hypomineralized, widened growth plates, increased metaphyseal cartilage remnants, fewer and smaller chondroclasts or osteoclasts, and altered MMP-9 localization (Miao et al., 2004). Because mineralization, chondrocyte survival, matrix composition, and resorptive-cell abundance change together, this phenotype cannot isolate resorption as the cause of trabecular disorganization. Across these models, the more defensible conclusion is conditional: delayed clearance increases persistent scaffold only when vascular and osteogenic access are preserved; when access fails, it arrests the cartilage-to-bone transition.

### 4.3 The proposed risk of premature scaffold loss remains under-tested

The converse prediction—that excessive or premature resorption removes longitudinal cores before osteoid deposition—has substantially weaker evidence. Osteoprotegerin deficiency increases RANKL availability and chondroclastogenesis, but the clearest cartilage data come from mouse fracture repair rather than native metaphyseal development. This model should be treated only as a mechanistic analogue: a transient fracture callus lacks the persistent longitudinal growth axis of the growth plate, experiences a different mechanical-loading regime, and is vascularized through a repair-specific route rather than the organized metaphyseal capillary front. In osteoprotegerin-deficient repair, cartilaginous callus resorption and union were accelerated rather than structurally impaired (Ota et al., 2009). The finding therefore shows that faster cartilage removal can be accommodated during repair, but it would generalize to primary trabecular formation only if accelerated resorption at the native growth plate were shown to precede osteoid coverage of longitudinal septa without a concomitant change in vascular route, matrix organization, or loading context.

Premature invasion can nevertheless alter skeletal form. In *Col9a1*-deficient mouse femoral heads, reduced antiangiogenic matrix signals coincided with early vascular and osteoclast invasion, accelerated ossification, and loss of normal epiphyseal anatomy (Heilig et al., 2020). The phenotype supports the importance of invasion timing, but it arises in a secondary ossification context and combines changes in matrix organization, angiogenic balance, and osteoclast entry. It therefore cannot demonstrate that excessive chondroclast resorption alone erased a required scaffold. Current evidence consequently establishes a lower bound for necessary scaffold retention more strongly than an upper bound: delayed cartilage clearance can be harmful, but premature chondroclast-mediated removal has not been isolated as a cause of abnormal primary trabecular geometry.

### 4.4 Early architectural errors can be corrected, but compensation has limits

Developmental timing defects are not invariably permanent. In *Mmp9*-null mice, delayed vascularization and ossification initially produced a greatly expanded growth plate, yet spontaneous compensatory remodeling after three postnatal weeks ultimately yielded an axial skeleton of normal gross appearance. The original report examined metatarsal growth plates from more than 50 mice across two genetic backgrounds, although the number analyzed at each age-specific endpoint was not reported (Vu et al., 1998). This observation therefore supports gross developmental compensation rather than demonstrated molecular or microarchitectural normalization. VEGF inhibition was likewise reversible: cessation of treatment restored capillary invasion, cartilage resorption, bone growth, and growth-plate organization (Gerber et al., 1999). Restoration of macrophage colony-stimulating factor in osteoclast-deficient *op/op* mice also recovered osteoclasts and removed abnormal diaphyseal trabeculae (Kodama et al., 1991). Together, these studies indicate substantial developmental plasticity, while distinguishing spontaneous compensation in the Mmp9-null model from recovery after restoration of a missing function while growth and remodeling remain active.

Gross recovery, however, does not prove normalization of trabecular topology, material composition, or mechanical performance. Later remodeling may remove retained cartilage and normalize bone length while preserving subtler differences in connectivity or regional shape. Conversely, the altered femoral-head anatomy in *Col9a1*-deficient mice indicates that mistimed invasion in a spatially constrained ossification center can coincide with persistent morphological change (Heilig et al., 2020). The available evidence therefore suggests that reversibility depends on the duration and anatomical location of the disturbance and on whether subsequent remodeling retains sufficient geometric information to reconstruct normal structure.

The resulting kinetic synthesis favors ordered overlap rather than maximal activity (Figure 1). Delayed entry can produce either inaccessible cartilage or cartilage-rich trabeculae depending on whether vascular and osteogenic access is preserved, whereas the architectural consequence of premature longitudinal-core loss remains unresolved. Across the available models, the principal unmeasured variable is the local lag among matrix loss, vessel occupancy, and osteoid coverage—not cartilage retention, vessel density, or bone volume considered in isolation.

## 5 Skeletal disorders expose distinct failure modes of the remodeling front

Cartilage retention, metaphyseal widening, and increased radiodensity are convergent outcomes rather than mechanism-specific diagnoses. They can reflect failure to generate resorptive cells, loss of function in cells that remain abundant, defective cartilage production, or a systemic mineral and endocrine disturbance. The models of osteopetrosis, hypophosphatemic rickets, and collagen-X proteostasis considered below therefore represent distinct causal-localization problems. Their initiating compartments, resorptive-cell phenotypes, mechanism-matched rescue evidence, and remaining interpretive boundaries are summarized in Table 2.

**TABLE 2:** Convergent cartilage retention arises from distinct initiating lesions.

Disease or model	Initiating lesion and compartment	Resorptive-cell phenotype	Mechanism-matched rescue evidence	What the evidence does not establish	References
TNFRSF11A (RANK)-deficient osteopetrosis	Receptor loss in hematopoietic osteoclast precursors	Osteoclast-poor	Five-patient case series: donor engraftment after HSCT in all patients; bone defects improved in three and remained under active remodeling in two at the reported follow-up, supporting hematopoietic-lineage replacement	Small cohort with heterogeneous follow-up (3.5 months–3 years); durability, consistency across TNFRSF11A variants, and a primary vascular effect remain unestablished	Pangrazio et al. (2012)
TNFSF11 (RANKL)-deficient osteopetrosis	Loss of a nonhematopoietic differentiation ligand	Osteoclast-poor	Systemic RANKL administration improved skeletal and marrow phenotypes in Rankl-null mice	Mouse ligand replacement is not validated as human therapy; HSCT does not replace the ligand-bearing niche	Lo Iacono et al. (2012)
TCIRG1- or SNX10-associated osteopetrosis	Osteoclast-intrinsic acidification or vesicular handling defect	Usually osteoclast-rich but ineffective	HSCT or gene-corrected hematopoietic strategies are mechanistically aligned with severe precursor-intrinsic disease	Clinical response and transplantation risk remain genotype-specific	Sobacchi et al. (2013), Donati et al. (2026)
CLCN7-, OSTM1-, or PLEKHM1-associated osteopetrosis	Ion transport or intracellular resorptive-machinery defects, with gene-specific extraskeletal effects	Commonly osteoclast-rich and dysfunctional	Molecular diagnosis determines whether hematopoietic replacement can address the lesion-bearing compartment	These genes are not interchangeable; neurological disease may remain outside an osteoclast-directed rescue	Sobacchi et al. (2013), Donati et al. (2026)
Ae2-null mouse	Osteoclast acidification defect	Present but functionally impaired	No mechanism-specific rescue has been established in the cited model	Broad cartilage plates and interwoven trabeculae demonstrate architectural consequences, not a human vascular mechanism	Josephsen et al. (2009)
Hyp mouse	PHEX loss with systemic FGF23–phosphate–endocrine disturbance	Secondary reduction in resorptive-cell number and activity	Phosphate manipulation and early 1,25-dihydroxyvitamin D treatment improved several skeletal outcomes	Multicompartment rescue cannot identify a primary local coupling defect	Hayashibara et al. (2007), Liu et al. (2016)
Mutant collagen-X ER-stress mouse model of Schmid metaphyseal chondrodysplasia	Hypertrophic-chondrocyte proteostasis and matrix-production defect	Downstream resorptive-cell state incompletely resolved	Enhanced intracellular proteolysis reduced ER stress and disease severity	The result does not generalize to every COL10A1 variant or identify the downstream vascular or resorptive mediator	Mullan et al. (2017)

Abbreviations: ER, endoplasmic reticulum; FGF23, fibroblast growth factor 23; HSCT, hematopoietic stem cell transplantation; PHEX, phosphate-regulating endopeptidase homolog, X-linked; RANK, receptor activator of nuclear factor-κB; RANKL, receptor activator of nuclear factor-κB ligand.

### 5.1 Osteopetrosis separates differentiation signals from cell-intrinsic execution

Autosomal recessive osteopetrosis provides the clearest human evidence that osteoclast abundance and matrix-removing capacity are distinct variables. TNFRSF11A (RANK) deficiency is intrinsic to hematopoietic osteoclast precursors and produces osteoclast-poor disease; In the five-patient case series cited here, all patients achieved donor engraftment after HSCT; bone defects had improved in three, whereas remodeling remained ongoing in two during follow-up ranging from 3.5 months to 3 years (Pangrazio et al., 2012). These outcomes are consistent with replacement of the defective lineage, but the small cohort and heterogeneous follow-up do not establish durable correction or uniform efficacy across variants.

Osteoclast-rich osteopetrosis requires genotype-level interpretation rather than one rescue rule. Defects in acidification or intracellular handling—commonly involving *TCIRG1*, *CLCN7*, *OSTM1*, *SNX10*, or *PLEKHM1*—permit multinucleated-cell formation but impair polarized resorption (Sobacchi et al., 2013; Donati et al., 2026). Human ultrastructure confirms that abundant osteoclasts can lack normal ruffled borders and clear zones and fail to resorb bone or cartilage (Shapiro et al., 1980). Several severe, osteoclast-intrinsic forms are candidates for HSCT, yet transplant responsiveness and extraskeletal risk differ by genotype; disease with primary neuronal involvement, for example, cannot be reduced to replacement of the resorptive lineage (Sobacchi et al., 2013; Donati et al., 2026). The common mechanistic lesson is narrower: radiodensity or tartrate-resistant acid phosphatase-positive cell abundance does not establish effective remodeling.

These disorders validate absent or ineffective mineralized-substrate resorption as a cause of defective cartilage-to-bone conversion. They do not establish vascular mispatterning as the primary lesion, because dense bone, retained cartilage, restricted marrow space, and vascular changes are generally assessed only after they have converged. Available studies report bone density and osteoclast abundance more consistently than cartilage-core persistence, marrow-space opening, vessel proximity, osteoid coverage, or trabecular orientation. The disease evidence therefore localizes defective resorption but does not reconstruct the complete matrix–vessel–bone sequence.

### 5.2 Hypophosphatemic rickets begins upstream of the local resorptive circuit

The Hyp mouse models phosphate-regulating endopeptidase homolog, X-linked (PHEX) deficiency and excess fibroblast growth factor 23 (FGF23)-driven phosphate wasting; the initiating lesion is not the cartilage matrix or osteoclast lineage itself. The resulting systemic mineral and endocrine environment then impairs cartilage mineralization and alters several front compartments. Hyp mice have widened hypomineralized growth plates, increased metaphyseal cartilage remnants, fewer and smaller chondroclasts or osteoclasts, and altered MMP-9 localization and hypertrophic-chondrocyte apoptosis (Miao et al., 2004). Retention therefore cannot be assigned solely to weak resorption because substrate properties, chondrocyte state, and recruitment cues change together.

Experimental phosphate manipulation supports a substantial environmental component. Low-phosphate conditions reduced osteoclast formation and resorption in culture and osteoclast abundance in mice, whereas phosphate repletion in Hyp mice improved osteogenesis and increased osteoclast abundance; bone marrow cells isolated from Hyp mice did not retain an intrinsic defect under standard culture conditions (Hayashibara et al., 2007). Early 1,25-dihydroxyvitamin D treatment also improved growth-plate and metaphyseal organization, trabecular microarchitecture, geometry, and strength without phosphate supplementation (Liu et al., 2016). Because this intervention altered serum phosphate, parathyroid hormone, renal transport, and chondrocyte behavior, it cannot be interpreted as selective rescue of resorptive–vascular coupling. Hyp mice instead show that a systemic lesion can generate a secondary remodeling-front phenotype.

### 5.3 A defined collagen-X proteostasis model propagates from chondrocytes

Metaphyseal chondrodysplasia type Schmid provides a chondrocyte-centered example, but the mechanistic inference should be limited to variants and models that cause intracellular collagen-X misfolding. In a mouse model expressing mutant collagen X with endoplasmic-reticulum retention, pharmacological enhancement of intracellular proteolysis reduced stress-marker expression and hypertrophic-zone expansion and improved longitudinal growth and skeletal dysplasia (Mullan et al., 2017). This rescue acts upstream of cartilage removal by correcting the state of the cell that builds the substrate and supplies local cues.

Reduced disease severity after correcting proteostasis establishes an important contribution of hypertrophic-chondrocyte stress in that model; it does not identify which downstream component mediates improvement. Altered collagen secretion can simultaneously change matrix topology, mineralization, protease programs, angiogenic-cue presentation, and osteoclast recruitment. The interface phenotype is therefore propagation from an upstream chondrocyte lesion, not evidence that all *COL10A1*-associated disease is primarily vascular or resorptive.

### 5.4 Mechanistic classification changes rescue logic

The same histological endpoint can encode different initiating lesions. RANK deficiency removes a hematopoietic receptor-dependent differentiation step; RANKL deficiency removes a nonhematopoietic ligand; osteoclast-rich osteopetrosis preserves multinucleated cells but disables their resorptive execution; PHEX deficiency first perturbs systemic phosphate regulation; and selected COL10A1 variants first disrupt hypertrophic-chondrocyte proteostasis. Consequently, osteoclast counts, bone volume, and hypertrophic-zone width cannot independently localize causality.

Existing rescue evidence illustrates the importance of matching an intervention to the lesion-bearing compartment. In the five-patient RANK-deficient case series, all patients achieved donor engraftment after HSCT; bone defects had improved in three, whereas remodeling remained ongoing in two at the reported follow-up, a pattern consistent with replacement of defective hematopoietic precursors (Pangrazio et al., 2012). RANKL administration instead addresses the missing nonhematopoietic ligand in a mouse proof-of-concept (Lo Iacono et al., 2012). Mineral/endocrine interventions in Hyp mice and proteostasis-directed treatment in the collagen-X model act on systemic or chondrocyte-centered initiating lesions, respectively (Liu et al., 2016; Mullan et al., 2017). Because these interventions operate at different causal layers and may correct several downstream processes simultaneously, improvement cannot by itself be attributed specifically to restoration of resorptive–vascular coupling.

Disease evidence therefore supports the remodeling front as a causal-localization framework rather than a single disease pathway. Direct human evidence for primary resorptive-cell–vascular uncoupling remains limited, and current disorders more clearly identify the first failed compartment than demonstrate restoration of the complete matrix–vessel–bone sequence. The experimental strategy required to distinguish primary coupling defects from secondary interface consequences is consolidated in Section 5.

## 6 Converting functional coupling into a testable causal model

The remodeling-front model becomes predictive only when experiments distinguish coexistence from directionality. Most perturbations alter several compartments and are read out after cartilage retention, vascular delay, and abnormal bone formation have converged. Four design requirements—temporal control, activity-resolved imaging, three-dimensional registration, and hierarchical endpoints—can separate direct coupling from shared upstream control (Figure 2), while a predefined prediction matrix links temporal ordering and rescue behavior to explicit model rejection (Figure 3).

**FIGURE 2:**
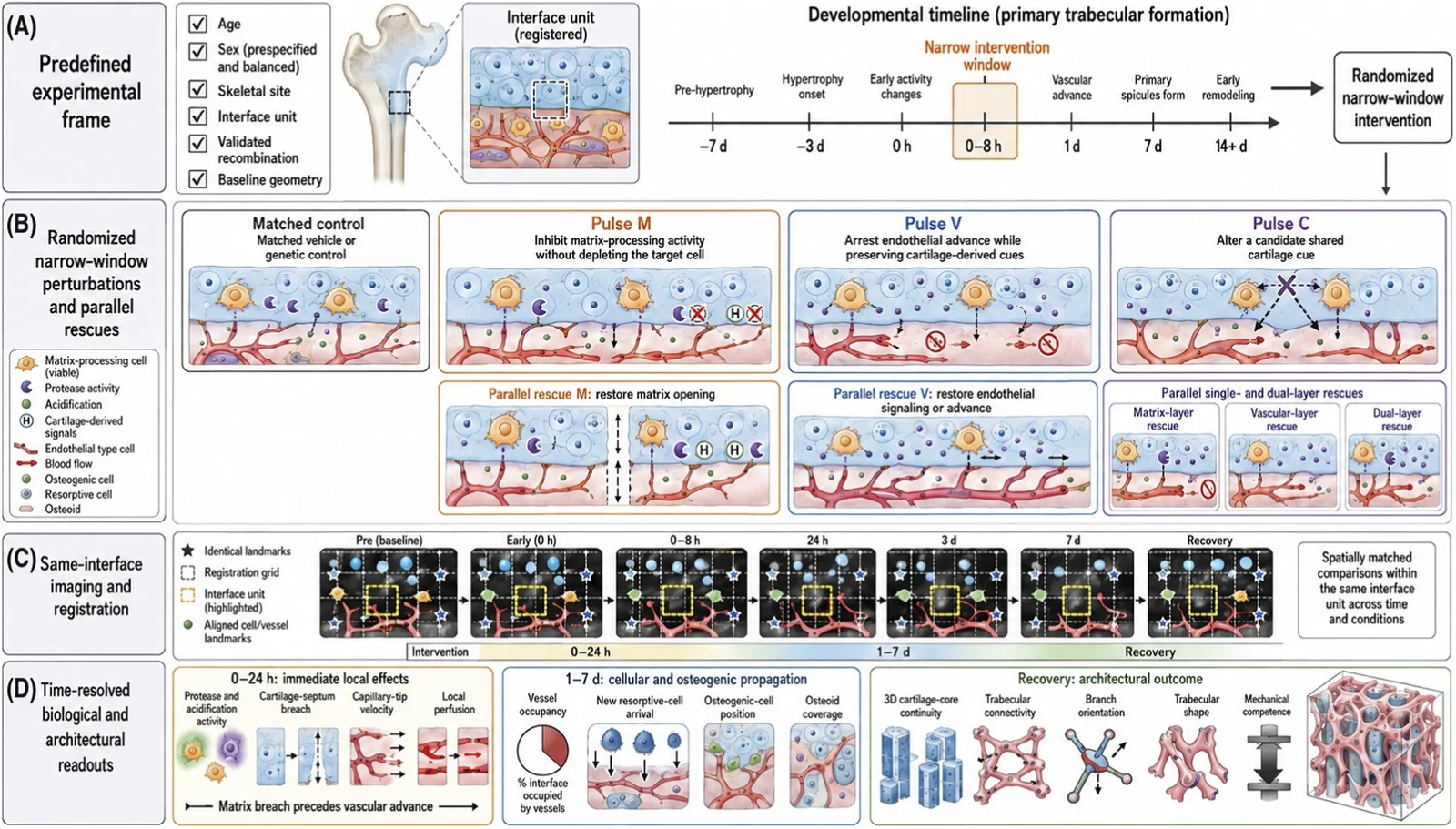
Registered narrow-window perturbations resolve the sequence from local intervention to architectural outcome. **(A)** The predefined experimental frame specifies developmental age, prespecified and balanced sex, skeletal site, registered interface unit, validated recombination, baseline geometry, and a 0–8-h intervention window. **(B)** Matched controls and randomized matrix-directed (Pulse M), vascular-directed (Pulse V), and shared-cue (Pulse C) perturbations are paired with single- and dual-layer rescues. **(C)** Repeated measurements are spatially registered to the same capillary-tip–cartilage interface unit across time and experimental conditions. **(D)** Time-resolved readouts separate immediate local effects at 0–24 h, cellular and osteogenic propagation at 1–7 days, and recovery-stage architectural outcomes. Signals that cannot be acquired simultaneously may be collected in compatible reduced panels and spatially co-registered.

**FIGURE 3:**
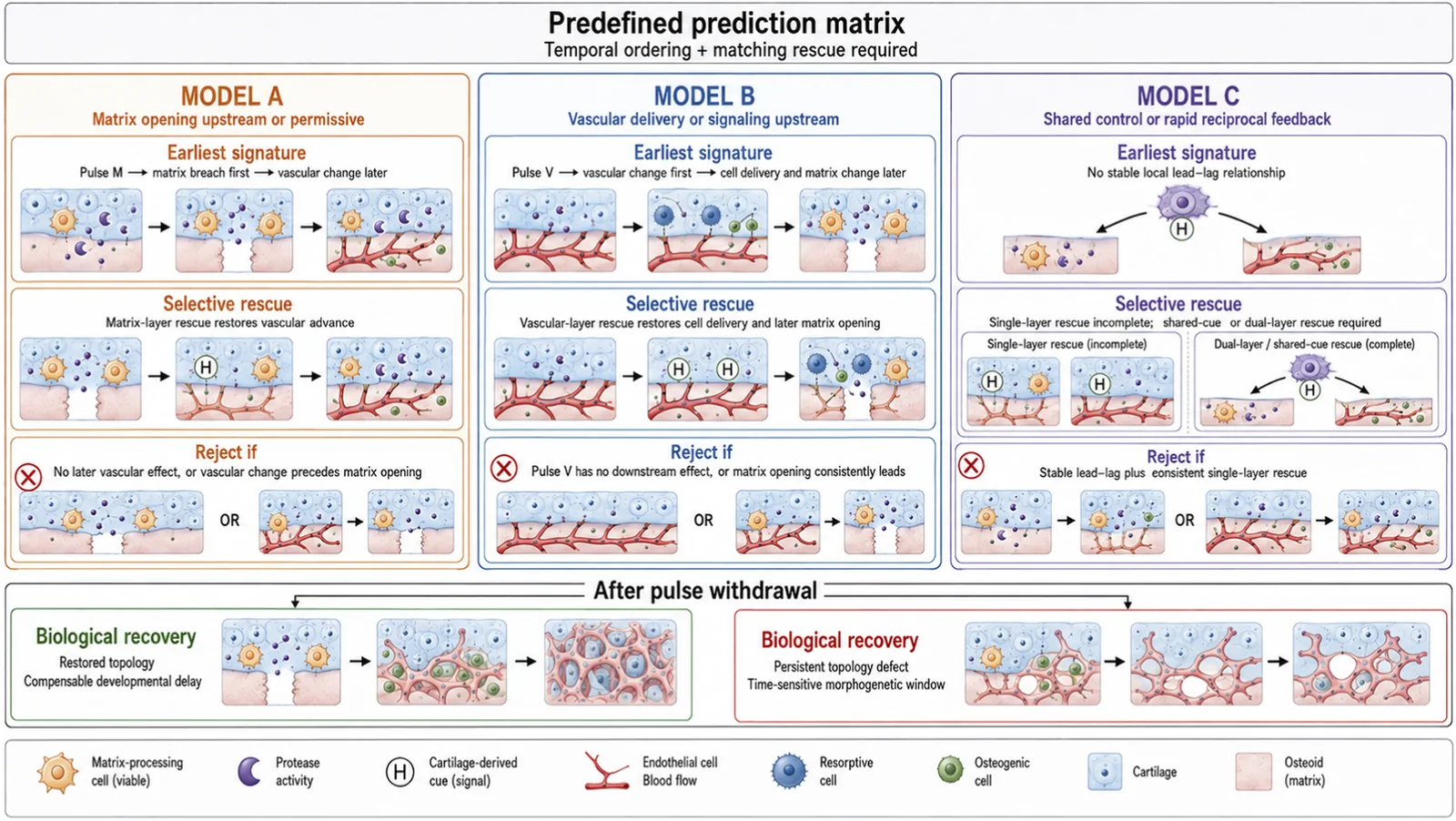
Predefined temporal and rescue signatures discriminate competing causal models. **(A)** Model A predicts that altered matrix opening precedes vascular change and is corrected by matrix-layer rescue. **(B)** Model B predicts that altered vascular delivery or signaling precedes changes in cell arrival and matrix processing and is corrected by vascular-layer rescue. **(C)** Model C predicts no stable local lead–lag relationship and incomplete correction by either single-layer rescue. Model assignment requires concordance between the prespecified temporal signature and rescue response; either criterion alone is insufficient. Following withdrawal of the pulse, restoration of both local biology and topology indicates a compensable delay, whereas biological recovery with persistent architectural abnormality identifies a time-sensitive morphogenetic window. These are proposed falsification criteria rather than a validated experimental algorithm.

### 6.1 Directionality requires temporally restricted and reciprocal perturbations

Constitutive gene deletion is poorly suited to ordering events at a moving interface. A gene altered in embryonic chondrocytes, endothelial cells, or myeloid precursors can change matrix composition, cell abundance, and tissue geometry before the first capillary reaches terminal cartilage. Even inducible Cre systems require validation of recombination timing, efficiency, and off-target activity; skeletal Cre lines frequently label broader or earlier populations than their shorthand names imply (Elefteriou and Couasnay, 2021). Experiments should therefore document the cells recombined at the moment of perturbation rather than infer specificity from the promoter alone. Sex is an additional unresolved design variable across this evidence base: the mouse studies reviewed here did not consistently report sex-stratified outcomes or test sex-by-genotype interactions, and their findings therefore cannot be assumed to be sex-invariant. Future pulse-perturbation studies should prespecify sex, include adequately represented females and males unless a developmental rationale justifies restriction, and test sex-by-perturbation interactions at the animal level rather than rely on underpowered *post hoc* subgroup comparisons.

A decisive design would perturb one compartment within a narrow developmental window and measure the other before secondary architectural changes emerge. Acute inhibition of resorptive activity should be followed within hours to days by measurements of transverse-septum opening, capillary-tip velocity, perfusion, and endothelial branching. To test the less-supported converse prediction, a matched gain-of-function pulse should transiently accelerate cartilage resorption while preserving endothelial and osteogenic programs, followed by registered measurements of capillary arrival, osteoid coverage, and nascent three-dimensional trabecular geometry. The reciprocal loss-of-function experiment should transiently arrest endothelial advance while preserving cartilage-derived signals and then determine whether resident resorptive cells remain positioned and active. If resorption is upstream, loss of matrix opening should precede vascular arrest; if precursor delivery is dominant, vascular arrest should reduce subsequent clast recruitment without immediately suppressing cells already present. A shared upstream signal would instead alter both processes without a consistent local lead–lag relationship. At the endothelial protease layer, matched pulse perturbations of Mmp9, Mmp14, and both genes within the same postnatal window would distinguish a uniquely required MMP-9 function from additive or compensatory proteolysis.

Rescue and epistasis are needed to move beyond temporal precedence. Restoring a matrix-opening activity in a vascular-deficient background would test whether physical access is sufficient, whereas restoring endothelial signaling without correcting an undegraded barrier would test whether angiogenic permission can overcome matrix obstruction. The interpretation must remain cell-specific: osteoclast-lineage drivers can address multinucleated clasts, but no equivalently selective, validated system yet isolates septoclasts. Until such a tool exists, loss of a broad myeloid or perivascular population cannot establish septoclast necessity.

In the near term, this limitation can be reduced without treating any single marker as septoclast-specific. Targeted spatial transcriptomics or multiplexed RNA/protein imaging at registered capillary-tip–septum units could combine Pdgfrb-lineage history with FABP5, ETS1, CTSB, MMP-13, and MMP-9, while excluding Vav1-lineage hematopoietic cells, multinucleated TRAP/CTSK-positive clasts, and endothelial cells (Sivaraj et al., 2022; Bando et al., 2025). Short-pulse lineage labeling and serial three-dimensional imaging could then test whether the appearance or loss of this composite state precedes transverse-septum removal and capillary advance; laser-capture or spatially indexed recovery from matched sites could nominate candidate effectors for local or intersectional perturbation. These approaches would reduce cell-state ambiguity and prioritize causal targets, but marker overlap and state plasticity mean that they would not, by themselves, prove exclusive septoclast necessity.

### 6.2 Cell identity must be separated from local resorptive activity

Static markers report potential, not execution. Tartrate-resistant acid phosphatase, cathepsin K, or multinucleation can identify osteoclast-lineage cells but cannot show whether a cell is polarized against cartilage, acidifying a resorption compartment, or degrading the specific septum adjacent to an advancing vessel. This distinction is clinically evident in osteoclast-rich osteopetrosis, where abundant cells can remain functionally ineffective (Shapiro et al., 1980; Sobacchi et al., 2013). It is equally important in developmental models in which cell number and matrix accessibility change together.

Functional probes provide a route forward. A cathepsin K-responsive two-photon probe has enabled real-time tracking of osteoclast activity in murine long-bone disease models and detection of pharmacological suppression without terminal tissue collection (Koo et al., 2024). This establishes feasibility, not yet direct visualization of chondroclast action at the developmental front. Cathepsin K activity also does not report acidification, substrate identity, or septoclast-mediated degradation. An informative imaging strategy would therefore seek complementary signals: vascular perfusion and endothelial membranes; local acidification or protease activity; mineralized and non-mineralized cartilage boundaries; and nascent osteoid. This list defines the required measurements rather than implying that all signals can be acquired simultaneously. Within a small, deep, and mineralized imaging volume, fluorophore compatibility, spectral overlap, excitation requirements, photon budget, and phototoxicity may restrict intravital acquisition to a reduced core panel, such as vascular position plus one activity reporter. Matrix boundaries and osteoid may then need to be acquired sequentially, in a separate spectral panel, or by registered endpoint imaging. Sequential datasets should be aligned using stable anatomical or exogenous fiducial landmarks, with both acquisition lag and spatial registration error quantified and incorporated into estimates of capillary-tip distance and event ordering. Whether acquired simultaneously or sequentially, activity should be registered to the same capillary tip and matrix element over time rather than compared as average signals across the metaphysis.

Long-bone depth, mineral scattering, motion, and the small size of the advancing front make repeated intravital imaging technically difficult. Short-term *ex vivo* bone cultures may offer controlled access, but removal from circulation alters flow, oxygenation, precursor delivery, and endocrine inputs. Concordance between intravital observations, *ex vivo* perturbations, and fixed-tissue time series would therefore be more persuasive than treating any one platform as a complete physiological representation.

### 6.3 Three-dimensional geometry must be linked to molecular state

Two-dimensional sections cannot reliably determine whether a cartilage remnant is an isolated plate, a longitudinal core, or part of a connected network; nor can they establish whether a vessel contacts, bypasses, or terminates beside that structure. Bone-clearing approaches such as Bone CLARITY preserve intact specimens for multiscale three-dimensional imaging (Treweek et al., 2021), and quantitative whole-bone platforms can map vessel phenotypes and skeletal progenitors at single-cell resolution (Rindone et al., 2021). The latter work also showed that osteoclast-lineage PDGF-BB perturbation weakened spatial associations between specialized vessels and progenitors in cranial bone (Rindone et al., 2021). This demonstrates analytical feasibility but cannot be transferred uncritically to endochondral long-bone development, because cranial osteogenic fronts lack the same cartilage topology.

For the chondro-osseous junction, three-dimensional analysis should segment at least four registered networks: calcified-cartilage cores, endothelial branches, resorptive-cell activity, and osteoid or mineral deposition. Relevant outcomes include capillary-tip distance from terminal septa, the lag between septum loss and vessel occupancy, cartilage-core orientation and continuity, osteoid coverage of retained cores, and eventual trabecular connectivity. These measurements would test the proposed timed handoff directly and avoid using total bone volume as a surrogate for ordered architecture.

Molecular profiling can identify the states that occupy these spatial positions, but dissociation removes precisely the matrix and vascular relationships central to the model. Single-cell RNA sequencing has reconstructed growth-plate chondrocyte maturation programs (Li et al., 2016), while combined inducible lineage tracing and transcriptomics indicate that hypertrophic chondrocytes can generate marrow-associated skeletal progenitors with vascular-recruiting properties (Long et al., 2022). These findings nominate candidate cell states and signals; they do not establish where or when those signals act at an intact front. Spatially resolved transcript or protein measurements should therefore be anchored to the imaged matrix–vessel geometry, followed by targeted perturbation of a small number of candidate interactions. Omics should generate causal tests rather than serve as the endpoint.

### 6.4 Causal success requires a hierarchy of structural endpoints

A mechanistic experiment should separate immediate, intermediate, and persistent consequences. Immediate endpoints include local matrix breach, resorptive activity, and capillary advance. Intermediate endpoints include vascular occupancy, osteogenic-cell recruitment, osteoid coverage, and the proportion of retained longitudinal versus transverse cartilage. Persistent endpoints include three-dimensional trabecular connectivity, marrow-space organization, bone length and shape, material composition, and mechanical performance. Measuring only the last tier obscures the initiating defect; measuring only the first cannot show morphogenetic significance.

Figure 2 provides an illustrative numerical implementation of this hierarchy. Following baseline registration, the perturbation would be restricted to 0–8 h, with the same interface units assessed at 24 h, 3 days, 7 days, and at a recovery endpoint of 14 days or later. A practical planning target would be 10–12 spatially separated capillary-tip–septum units per animal, sampled across at least three nonadjacent regions, with a prespecified minimum of eight successfully registered units retained through the 7-day assessment. These interface units are repeated subsamples rather than independent biological replicates; the animal remains the experimental unit. For each registered unit (t_M_) and (t_V_) would denote the first detected matrix-breach or protease change and the first detected vascular-perfusion or advance change, respectively, with the lead–lag interval defined as (L = t_V_-t_M_). Repeated measurements should be analyzed using a mixed-effects model containing perturbation, rescue, time, and their interactions as fixed effects, with interface unit nested within animal as random effects.

Model assignment should combine the direction of (L) with the prespecified rescue pattern rather than rely on separate endpoint-wise *P* values. Using the 0–8-h intervention window as an operational equivalence margin, Model A would require the confidence interval for (L) to lie above +8 h, an early effect of Pulse M followed by vascular delay, and normalization by matrix-layer rescue. Model B would require the interval to lie below −8 h, an early effect of Pulse V followed by matrix or resorptive-cell change, and normalization by vascular-layer rescue. Model C would require statistical equivalence within ±8 h, or simultaneous changes during the earliest sampled interval, together with failure of either single-layer rescue and restoration by a shared-cue or dual-layer rescue. A confidence interval that merely crosses zero would remain inconclusive rather than be assigned to Model C. Because platform- and interface-specific imaging variance has not yet been established, the definitive animal number should be based on pilot-derived between-animal variance. As a worked planning example, detecting an 8-h between-arm shift in animal-level (L) with two-sided α = 0.05 and 80% power would require approximately 10 animals per arm if the standard deviation were 6 h, or 17 animals per arm if it were 8 h.

The strongest test would combine a pulse perturbation with longitudinal recovery. Transient-window models are not intended to reproduce the duration or distribution of a human systemic mutation. Their translational value is to identify when a persistent lesion first becomes architecture-setting and which downstream operations remain reversible. Applying brief perturbations or mechanism-matched rescues at successive ages within a constitutive disease background could distinguish a sensitive window from a compensable delay and an irreversible defect. In chronic RANK/RANKL, PHEX, or collagen-X disorders (Table 2), such maps could indicate when correction of the initiating lesion is essential and when a downstream local rescue might still normalize matrix–vessel–bone progression, while also nominating stage-specific structural biomarkers. Pulse models therefore translate chronic disease into a temporal map of causal vulnerability and rescue opportunity rather than a literal model of transient clinical exposure. If a brief delay shifts vessel entry and cartilage retention but later remodeling restores topology, the coupling mechanism controls developmental timing without permanently specifying architecture. If a transient perturbation leaves a persistent branching or shape defect after cellular and molecular readouts normalize, the front contains a time-sensitive morphogenetic window. Comparing these outcomes across primary and secondary ossification centers would further test whether reversibility depends on anatomical context rather than on the molecular lesion alone.

The immediate priority is a registered causal sequence in one tractable long-bone model, not a broader atlas. A minimally decisive experiment would perturb one compartment within a narrow window, image matrix degradation and vascular advance at the same interface, reconstruct the resulting cartilage–bone network, and rescue the adjacent causal layer (Figure 2). The resulting trajectories should then be evaluated against prespecified temporal and rescue criteria to distinguish matrix-first, vascular-first, and shared-control models (Figure 3).

## 7 Conclusion: remodeling is a morphogenetic operation at the cartilage-to-bone boundary

Early skeletal remodeling is not merely post-patterning disposal of a cartilage template. At the chondro-osseous junction, transverse cartilage is preferentially processed near advancing vessels, longitudinal calcified septa remain as temporary osteoid-bearing cores, and later remodeling removes those cores (Lee et al., 1995; Mackie et al., 2008; Amizuka et al., 2012). These observations motivate—but do not yet prove—a morphogenetic timing rule in which the position and lag of matrix processing, vascular occupation, and osteoid deposition influence primary trabecular architecture.

Current evidence replaces a simple “clast clears, vessel follows” account with several stage- and cell-state-specific dependencies. VEGF-A blockade demonstrates that angiogenic entry is required for coordinated cartilage remodeling (Gerber et al., 1999). Perlecan rescue shows that matrix presentation conditions endothelial VEGF signaling (Ishijima et al., 2012). Endothelial MMP-9 contributes directly to cartilage processing, while vessel-associated osteoclasts regulate type H vessel anastomosis (Romeo et al., 2019). Endothelial Dll4 supports a mesenchymal septoclast state (Sivaraj et al., 2022). Endothelial Notch signaling also supports osteoprogenitor abundance and osteogenesis (Ramasamy et al., 2014). Classical descriptions identify osteogenic cells associated with vascular entry (Mackie et al., 2008), while independent lineage-tracing studies identify an additional contribution from hypertrophic-chondrocyte descendants (Yang et al., 2014; Zhou et al., 2014). Osteoclast-supporting RANKL is likewise distributed rather than fixed: hypertrophic-chondrocyte Piezo1 regulates the local RANKL/OPG balance in defined postnatal and repair contexts (Tschaffon-Müller et al., 2026), whereas fetal CD55^+^CD90^+^ interface mesenchyme and later LepR-positive marrow stroma form a developmental relay (Sumiya et al., 2026). These findings strengthen a multicompartment framework while making any universal linear circuit less defensible.

This distinction also changes how convergent disease phenotypes should be interpreted. RANK- and RANKL-deficient osteoclast-poor osteopetrosis, osteoclast-rich defects of resorptive execution, PHEX-related systemic mineral disturbance, and defined collagen-X proteostasis defects can all produce cartilage retention while failing at different points in the matrix–vessel–bone sequence (Table 2). Recovery is therefore better defined by restoration of the ordered fate of a cartilage element—matrix opening, vascular occupancy, osteoid coverage, and subsequent core removal—than by normalization of cartilage abundance or bone volume alone. This element-level criterion may ultimately distinguish a recoverable developmental delay from an early geometric error that later remodeling cannot erase.
